# Supplementary material for: Integrative Single-Cell and Bulk RNA Sequencing Identifies a Glycolysis-Related Prognostic Signature for Predicting Prognosis in Pancreatic Cancer
Source: Int J Mol Sci. 2025 May 26;26(11):5105. doi: 10.3390/ijms26115105 (PMC12154285; doi:10.3390/ijms26115105)
Supplement: Supplementary file 1 [file ijms-26-05105-s001.zip › ijms-3601508-supplementary.pdf]

Gene	HR	lower 95%CI	upper 95%CI	pvalue
NUP37	2.660	1.497	4.728	0.001
NDC1	2.430	1.553	3.803	0.000
NUP35	2.193	1.092	4.406	0.027
NUP93	2.141	1.078	4.251	0.030
NUP107	1.877	1.100	3.203	0.024
NUP160	1.760	1.048	2.954	0.033
ENO1	1.643	1.209	2.233	0.002
PGM2L1	1.594	1.167	2.179	0.003
HK1	1.557	1.094	2.215	0.015
PGK1	1.503	1.099	2.057	0.011
PKM	1.426	1.045	1.946	0.026
HK2	1.299	1.098	1.536	0.002
GCKR	1.264	1.017	1.572	0.034
GCK	0.676	0.505	0.905	0.007
PFKM	0.617	0.421	0.904	0.014
RAE1	0.522	0.276	0.987	0.047
PRKACA	0.507	0.310	0.832	0.008
NUP88	0.505	0.285	0.896	0.019
AAAS	0.499	0.296	0.842	0.009
POM121C	0.481	0.275	0.841	0.011
ENO3	0.452	0.249	0.823	0.009
NUP214	0.434	0.237	0.796	0.008
G6PC3	0.430	0.264	0.701	0.001
NUP42	0.416	0.251	0.691	0.001
SLC25A11	0.288	0.166	0.499	0.000

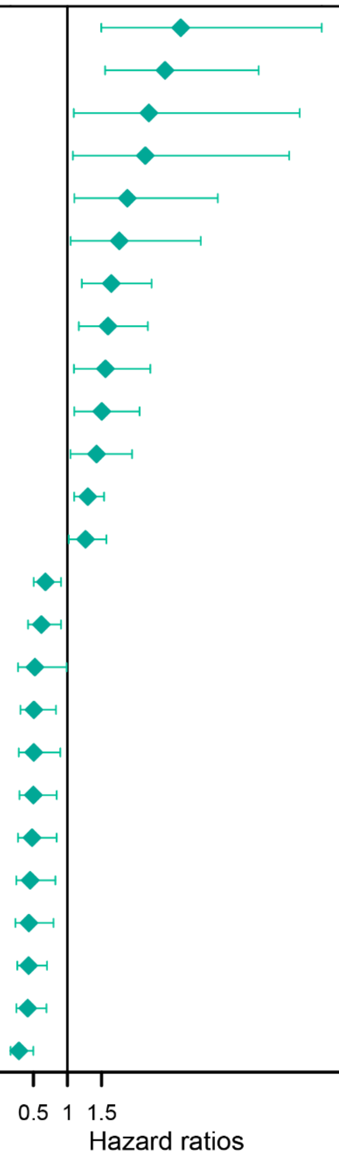

Supplement Figure S1 25 glucose metabolism related prognostic genes generated from univariable Cox analysis.

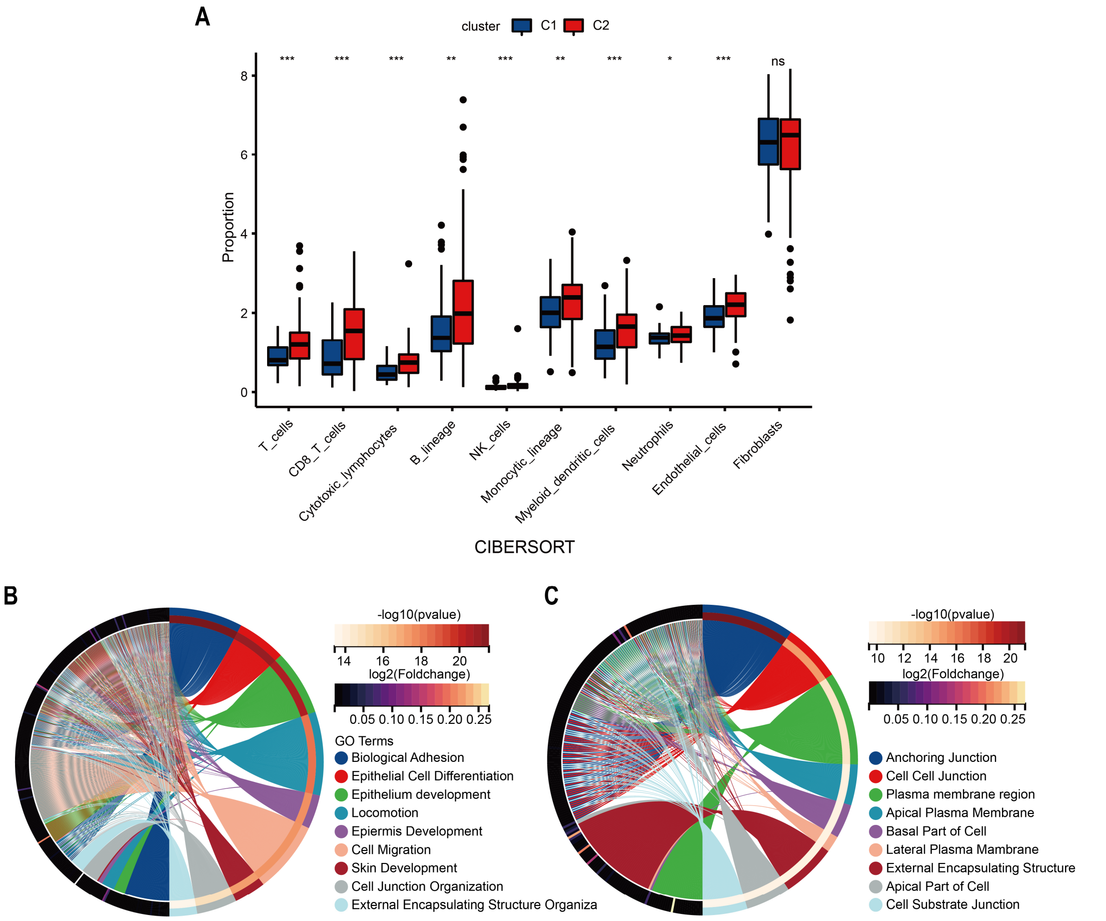

Supplement Figure S2 A The enriching level of 10 immune related cells evaluated by CIBERSORT algorithm. **B, C** The biological processes (BP) and cellular components (CC) of GO analysis.

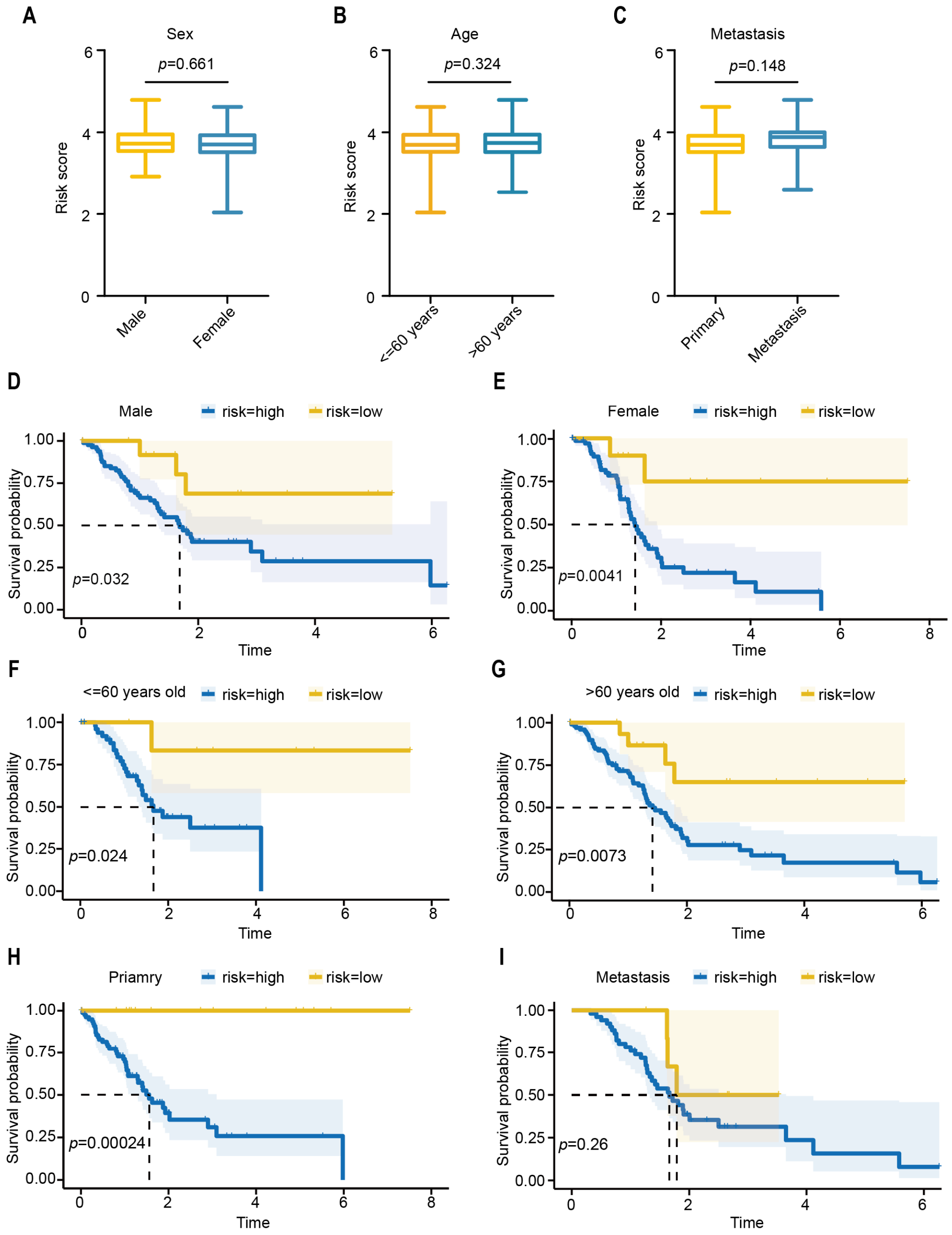

Supplement Figure S3 The association between the risk score and clinical features.

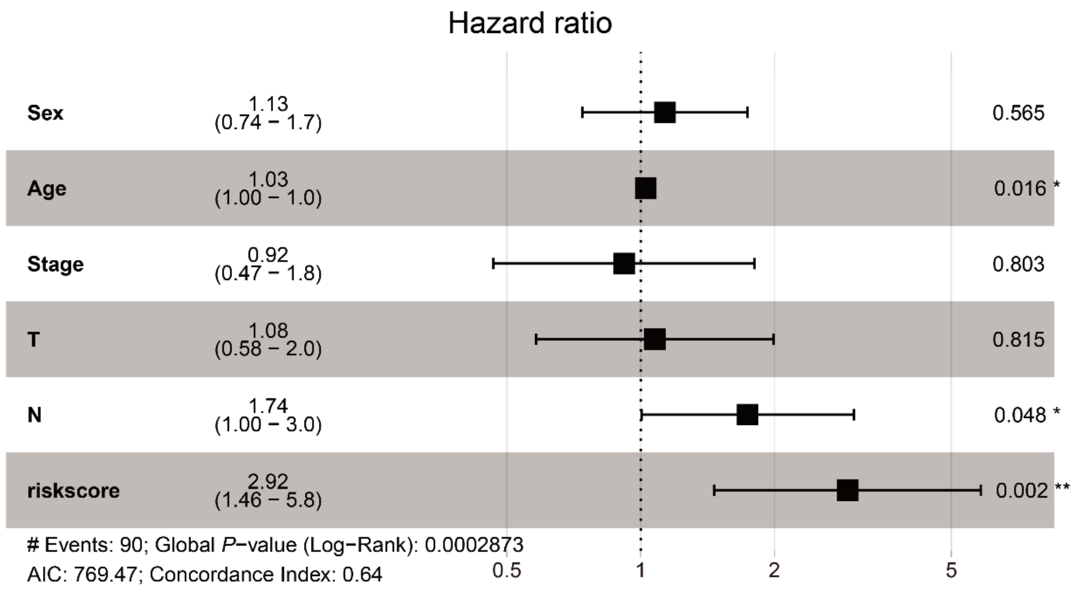

Supplement Figure S4 Multivariate Cox analysis in the TCGA cohort. (* $p < 0.05$; ** $p < 0.01$; Cox proportional-hazards regression test)

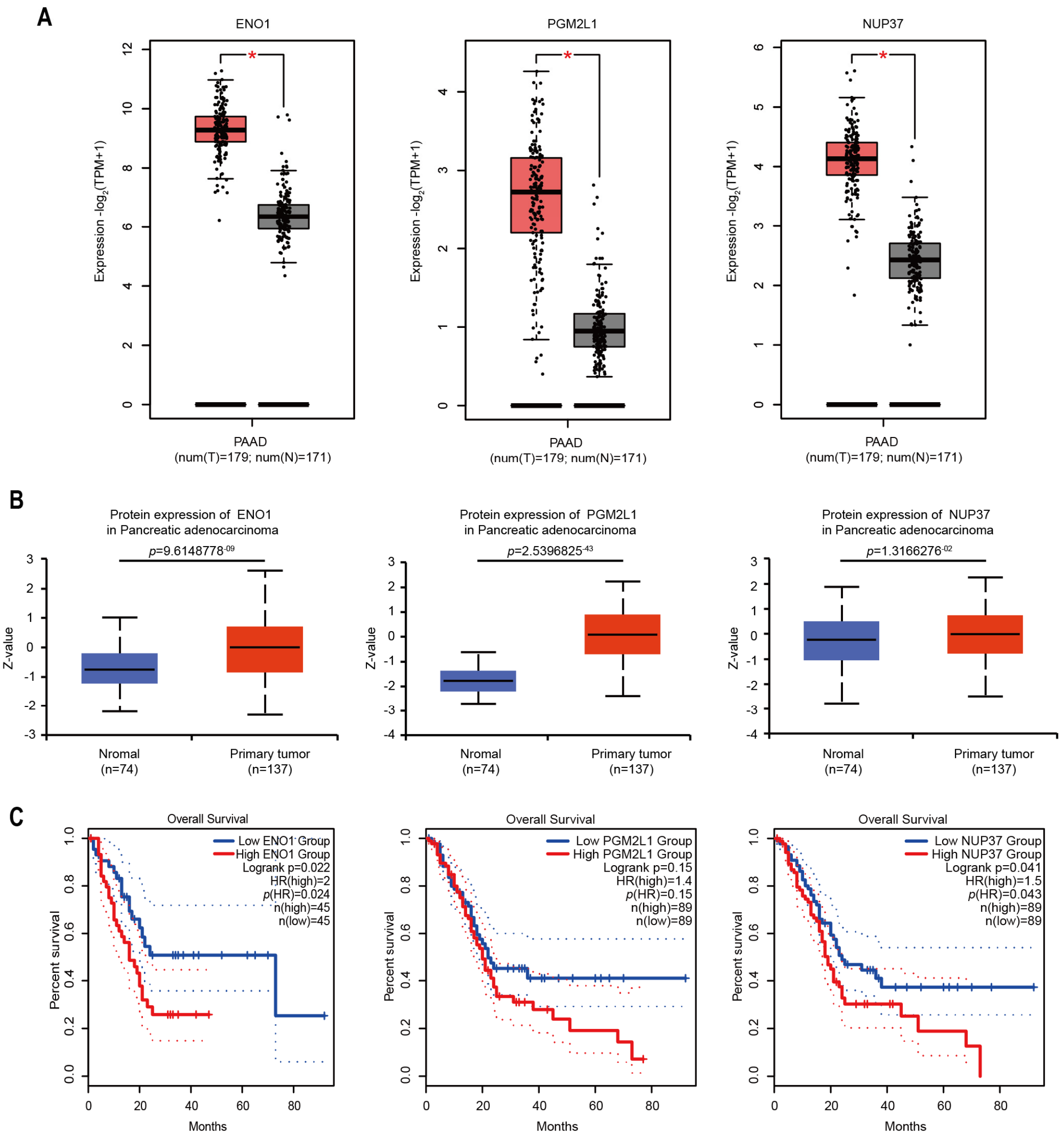

Supplement Figure S5 The expression levels of mRNA (A) and protein (B) of ENO1, PGM2L1 and NUP37. C Survival curve of ENO1, PGM2L1 and NUP37 in TCGA cohort.

A

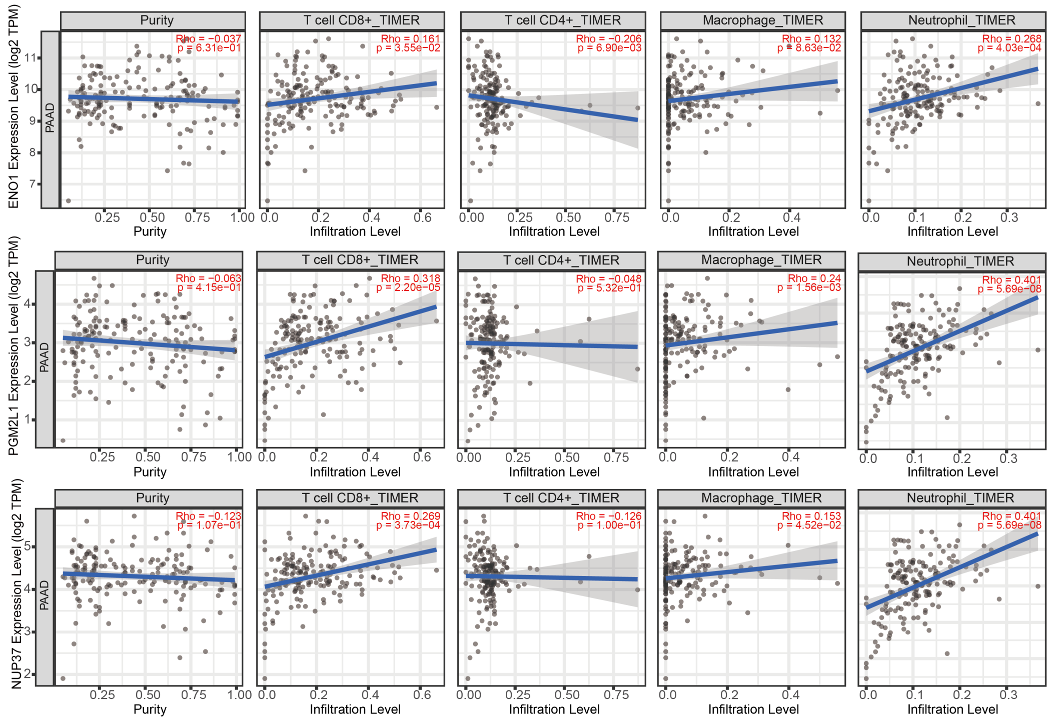

Supplement Figure S6 Correlations of ENO1, PGM2L1 and NUP37 expression and immune cell infiltration level in PAAD.
